# IGF signalling in germ cells and testicular germ cell tumours: roles and therapeutic approaches

**DOI:** 10.1111/andr.12658

**Published:** 2019-06-09

**Authors:** J. Selfe, J. M. Shipley

**Affiliations:** ^1^ Sarcoma Molecular Pathology Team Divisions of Molecular Pathology and Cancer Therapeutics The Institute of Cancer Research London UK

**Keywords:** AKT, cancer, insulin‐like growth factor, insulin‐like growth factor 1 receptor, testicular germ cell tumour, testis cancer

## Abstract

The insulin‐like growth factor (IGF) axis plays key roles in normal tissue growth and development as well as in the progression of several tumour types and their subsequent growth and progression to a metastatic phenotype. This review explores the role of IGF system in normal germ cell development and function in addition to examining the evidence for deregulation of IGF signalling in cancer, with particular relevance to evidence supporting a role in testicular germ cell tumours (TGCTs). Despite the clear preclinical rationale for targeting the IGF axis in cancer, there has been a lack of progress in identifying which patients may benefit from such therapy. Future employment of agents targeting the IGF pathway is expected to concentrate on their use in combination with other treatments to prevent resistance and exploit their potential as chemo‐ and radiosensitizers.

## Introduction

**Figure 1:**
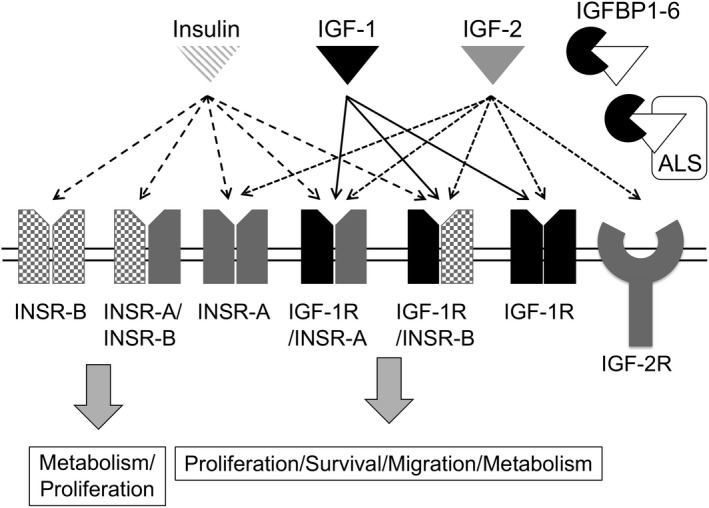
Insulin and IGF ligand specificity for INSR/IGF1R receptors. Each of the insulin receptors INSR‐A, INSR‐B and IGF1R transcripts encodes a single polypeptide chain, which undergoes proteolytic cleavage to produce an α and β subunit. Each αβ subunit forms homo‐ or heterotetramers. Insulin and the IGF ligands can bind competitively to these receptors as shown. Bioavailability of IGF ligands is regulated by insulin‐like growth factor binding proteins 1–6 (IGFBPs). IGFBPs bound to ligands can also bind the acid labile subunit (ALS) in the bloodstream to form a ternary complex which is thought to be unable to cross the capillary endothelium unless partially dissociated. Insulin signalling primarily affects glucose metabolism (but can also regulate other functions including growth), signalling through the INSR‐A homotetramer or any receptor containing IGF1R subunits results in downstream activation of pathways involved in proliferation, prevention of apoptosis and migration. The structurally unrelated IGF2R receptor binds IGF2, the functional significance of which is yet to be fully established but may act to regulate extracellular IGF2 levels.

The insulin and insulin‐like growth factor (IGF) signalling system has been implicated in a vast array of both physiological and pathological cellular processes. The IGF family principally comprises three ligands (insulin, IGF1 and IGF2), three receptors [the insulin receptor (IR), the insulin‐like growth factor 1 receptor (IGF1R) and the insulin‐like growth factor 2 receptor (IGF2R)] in addition to six high affinity ligand binding proteins (IGFBP1‐6), and accessory proteins such as IGFBP‐specific proteases. The signalling through IR/IGFR is complex (see Fig. 1). IR and IGF1R are tyrosine kinase receptors that share a high degree of structural homology and can exist as either homo‐ or heterotetramers (hybrid receptors). Additional complexity arises through alternative splicing of the *INSR* gene resulting in two IR subunits IR‐A and IR‐B, which have differing affinities for insulin and the IGF ligands (Seino & Bell, 1989). This results in a total of seven different receptors. Insulin can signal through any receptor containing at least one IR subunit while IGF1 can signal through any receptor containing at least one IGF1R subunit. IGF2 can signal through the same receptors as IGF1 in addition to the IR‐A homotetramer as well as the IGF2R homodimer (reviewed in Simpson *et al*., 2017). IGF2R is structurally unrelated to IR/IGF1R and possesses no tyrosine kinase activity or autophosphorylation sites. The IGF2R receptor can bind to G‐proteins, however, and despite previous assertions that IGF2R functions only to control IGF2 ligand levels by acting as a sink receptor, it is possible that it can initiate downstream signalling (El‐Shewy *et al*., 2007). While signalling through the IR‐A/B and IR‐B receptors mainly contributes to glucose homeostasis, signalling through the IGF1R hetero‐ and homotetramers generally leads to activation of both anti‐apoptotic mechanisms resulting in increased cell survival and increased cellular proliferation and growth in normal and malignant tissues.

## Role of IGF System in Testicular Function: Lessons Learned from Genetic Models

Signalling through the insulin and Igfr receptors are absolutely required for sex determination in mice (Nef *et al*., 2003). There is complete XY sex reversal due to failure to upregulate *Sry* and a complete failure of the testicular genetic programme in mouse embryos with homozygous deletion of the *Insr* and *Igf1r* genes. Ovarian differentiation is also delayed in XX gonads in the same model, although the ovarian genetic programme is eventually initiated several days later than in control embryos (Pitetti *et al*., 2013a). It has subsequently been shown that specific ablation of the *Insr* and *Igf1r* genes in Sertoli cells drastically influences testis size, Sertoli cell number and sperm production, whereas deletion of these genes in just the germ cells themselves results in normal testicular function and size (Pitetti *et al*., 2013b). The same study showed that a disruption to the neonatal action of follicle‐stimulating hormone (FSH) on immature Sertoli cells occurred in the absence of Insr/Igf1r (IIR) signalling. A more recent study concluded that although foetal Leydig cell function is normal, adult Leydig cells fail to mature using the constitutive double knockout model (Neirijnck *et al*., 2018).

IGF1 has reversed testicular atrophy induced by cirrhosis of the liver in rats (in which IGF1 levels are reduced), resulting in full recovery of testicular weight and reversal of all histopathological abnormalities (Castilla‐Cortazar *et al*., 2000). Although specific abrogation of IIR signalling in germ cells did not appear to impair testicular function in mice, there is evidence that germ cell function is profoundly affected by IGF signalling. In keeping with the recognized role of IGF1 as an inhibitor of apoptosis, in vitro organ culture of mouse testicular fragments supplemented with IGF1 increased the density of germ cells by decreasing apoptosis (Yao *et al*., 2017). Mice carrying a transgene‐expressing IGFBP1 exhibited defects in spermatogenesis with altered production and quality of spermatozoa, attributed to the lack of bio‐available IGF1 caused by increased binding of IGFBP1 (Froment *et al*., 2004). IGF1 administration was able to reverse the decrease in germ cell numbers observed in rats with surgically induced unilateral undescended testes (Bingol‐Kologlu *et al*., 2010). Co‐culture of Leydig cells with mouse spermatogonial stem cells (SSCs) and subsequent blockade of IIR signalling led to loss of expression of pluripotent genes in SSCs, supporting the idea that IGF1 produced by Leydig cells can maintain SSC pluripotency (Huang *et al*., 2009). IGF1 has also been shown to influence steroidogenesis in cultured Leydig cells (Lin *et al*., 1986). Insulin receptor substrate 2 (IRS2) is one of the key downstream effectors of IIR signalling and has itself been implicated in testicular development. Mice with homozygous deletion of the IRS2 have reduced testicular weight with lower numbers of Sertoli cells, spermatogonia and spermatocytes (Griffeth *et al*., 2013).

In a zebrafish model, either ectopic overexpression of IGF‐I or dominant negative expression of IGF receptors in primordial germ cells (the putative cells of origin in TGCT) leads to defects in migration of these cells to the genital ridge (Sang *et al*., 2008). A separate study found that knocking down the IGFR1b gene in zebrafish embryos resulted in both mismigration and elimination of primordial germ cells (Schlueter *et al*., 2007). In *C.elegans*, mutations in the *daf‐2* gene (the single gene encoding an insulin/insulin‐like growth factor receptor in this species) led to infertility (Tissenbaum & Ruvkun, 1998) and a study using a conditional *daf‐2* allele demonstrated the necessity of IIR signalling for larval germ cell proliferation by promoting cell cycle progression (Michaelson *et al*., 2010). Together, these studies provide evidence for an essential role of IGF signalling in supporting the normal development of germ cells.

## Role of IGF System in Cancer Risk

There are several lines of long‐standing evidence linking higher serum levels of IGF1 (associated with increased growth) and decreased serum levels of some IGFBPs (associated with suppressing growth through binding IGF1) with additional cancer risk (reviewed in Crowe *et al*., 2011). The congenital overgrowth disorder, Beckwith–Wiedemann syndrome, is associated with increased cancer risk and is frequently associated with disrupted imprinting of the *IGF2* gene. The gene encoding the potent mitogen IGF2 is imprinted in normal somatic cells, with only the paternal allele being expressed. Loss of imprinting in this chromosomal location results in increased IGF2 levels by being biallelically expressed (Mussa *et al*., 2016).

It is noted that both height and body mass index (BMI) correlate with higher cancer risk and with increased circulating IGF1 levels and/or decreased IGFBP levels (Nunney, 2018). Consistent with these findings, patients with congenital secondary IGF1 deficiency are less likely to develop cancer (Steuerman *et al*., 2011), while mice with reduced circulating Igf1 levels experience delayed onset of mammary tumours compared to controls (Wu *et al*., 2003). In accordance with other tumour types, height has also been reported as a risk factor for testicular germ cell tumours (TGCTs) (Rasmussen *et al*., 2003; Richiardi *et al*., 2003; McGlynn *et al*., 2007), although there is no direct evidence that circulating IGF1 levels are linked to a higher risk of developing TGCT. However, the chromosomal disorder Klinefelter syndrome (47 XXY) is associated with increased height (Aksglaede *et al*., 2008) and an increased risk of mediastinal germ cell tumours (Nichols *et al*., 1987) but serum IGF1 and IGFBP3 levels in the normal range (Aksglaede *et al*., 2008).

Mice with elevated growth hormone (GH)/Igf1 serum concentration had a higher incidence and reduced latency of mammary tumours but only in the context of a high fat diet (Gahete *et al*., 2014). Modulating factors such as diet perhaps explains the lack of concordance in the literature when trying to assess the proportion of risk attributable to circulating IGF1 concentration and these factors may explicate the lack of such a relationship in TGCT. A large meta‐analysis examining the effects of circulating IGF1 and IGFBP3 levels on the risk of developing several common cancers detected an association between increased IGF1 concentration and prostate, colorectal and pre‐menopausal breast cancer risk, while perhaps surprisingly, *increased* IGFBP3 levels were associated with risk of pre‐menopausal breast cancer. This finding challenges the assumption that IGFBP3 only exerts its effects on cancer risk by regulating bioavailability of IGF1. This study did not detect a protective effect of lower IGFBP3 levels overall; however, when one of the lung cancer cohorts was removed (that recruited only heavy smokers and asbestos workers), the risk of lung cancer was significantly decreased in individuals with higher IGFBP3 concentration (Renehan *et al*., 2004). Overall, serum levels of IGF ligands have a modest effect on cancer risk and may need very large association or meta‐studies to detect them. An alternative mechanism for IIR activity to influence cancer risk would be altered expression of the IGF1R receptor in the target organ. In this regard, it is interesting to note that Igf1r concentration was higher in cryptorchid than normal testes post‐puberty in an induced rat model (Antich *et al*., 1995). Cryptorchidism is a well‐known risk factor for TGCT (Banks *et al*., 2013); however, the status of IGF1R expression is unknown in this condition in humans. Polymorphisms within IGF‐related genes have also been associated with risk of several cancers including breast and prostate although these are not necessarily linked to differences in circulating IGF levels (Al‐Zahrani *et al*., 2006; Canzian *et al*., 2006; Cao *et al*., 2014a; Jung *et al*., 2017). There is, however, no positive evidence linking polymorphisms in IGF genes to testicular cancer risk (Chia *et al*., 2008; Loveday *et al*., 2018).

## Role of IGF System in Oncogenesis

Increased expression of many components of the IGF family has been invoked in tumourigenic mechanisms. IGF1R, IGF1 and IGF2 are frequently overexpressed in a large number of tumour types (Papa *et al*., 1993; Bergmann *et al*., 1995; Sekyi‐Otu *et al*., 1995; Steller *et al*., 1996; Weber *et al*., 2002). Insulin‐like growth factor 2 mRNA binding proteins (IMPs) are expressed during embryogenesis and less so in normal adult tissues; however, they are upregulated in a broad range of cancers where their expression correlates with poor prognosis (reviewed in Degrauwe *et al*., 2016). Moreover, expression of IGF1R has been shown to be prerequisite for transformation by several different oncogenes (Sell *et al*., 1993; Toretsky *et al*., 1997). Several members of the IGF family are potentially dysregulated in TGCT. IGF1 and IGFBP5 are frequently expressed in the precursor TGCT lesion, germ cell neoplasia *in situ* (Drescher *et al*., 1997). Large‐scale de novo demethylation takes place in primordial germ cells, relaxing imprinting at most genomic locations. TGCTs frequently retain this loss of imprinting, expressing IGF2 biallelically (Van Gurp *et al*., 1994), which has been linked to increased tumour aggressiveness in other cancer types (Damaschke *et al*., 2017). Increased serum levels of IGF2 and IGFBP2 have been found in non‐seminomatous TGCT, decreasing upon successful therapy and increasing again in cases of recurrence (Fottner *et al*., 2008). Our group has recently shown that IGF1R is expressed in approximately half of non‐seminomas and influences survival of non‐seminoma cells *in vitro* (Selfe *et al*., 2018).

The IGF axis has been implicated in a wide number of oncogenic processes. Signalling through the IGF1R receptor primarily activates the PI3K/AKT and MAPK (Ras/Raf/MEK/ERK) pathways. Whereas activation of the MAPK pathway drives cellular proliferation through promoting proteins involved in cell cycle progression, signalling via the AKT pathway both activates anti‐apoptotic proteins and inhibits anti‐apoptotic proteins to enhance cell survival (Chitnis *et al*., 2008). Our study in TGCT cell lines suggested that these cells primarily signal through the PI3K/AKT pathway in response to IGF ligand, perhaps reflecting the activation of the MAPK pathway via other means such as the tyrosine kinase receptor *KIT* and *RAS* mutation or overexpression (McIntyre *et al*., 2004, 2005). IGF2 can rescue a teratocarcinoma cell line from undergoing apoptosis in the absence of serum (Engström, 2010), reinforcing the anti‐apoptotic properties of IIR signalling in the context of TGCT.

IGF1R signalling has also been associated with several cellular processes that contribute to metastasis. Migration and invasion have been linked to IGF1R activity through co‐operation with the integrin pathway leading to Rho‐A‐dependent motility via FAK and RACK1 (Doerr & Jones, 1996; Brooks *et al*., 1997; Zhang *et al*., 2005; Montagnani Marelli *et al*., 2006). The chemokine receptor CXCR4 (Goddard *et al*., 2007; Gilbert *et al*., 2009) is reported to be involved in the survival and migration of TGCTs as well as primordial germ cells (reviewed in Gilbert *et al*., 2011a). Notably, IGF1 signalling through IGF1R has been shown to increase migration and CXCR4 expression in both mesenchymal stem cells and embryonic germline stem cells (Li *et al*., 2007; Kuo *et al*., 2018).

Matrix metalloproteinases are induced by IGF1 (Yoon & Hurta, 2001), conferring an invasive phenotype (Das *et al*., 2018), and MMP‐2 and MMP‐9 are frequently expressed in non‐seminomas (Gilbert *et al*., 2011b). IGF1 can also induce VEGF ligands and upregulate vascular vessel formation, thereby exhibiting pro‐angiogenic properties (Kurmasheva *et al*., 2009; Li *et al*., 2011). IGF1R signalling appears to be required for epithelial‐to‐mesenchyme transition in some cancer cells (Graham *et al*., 2008; Yi *et al*., 2018), driving malignant progression. It is perhaps, therefore, unsurprising that IGF axis proteins have been linked to patient outcome (Kawamoto *et al*., 1998; Fu *et al*., 2011; Turney *et al*., 2011; Unger *et al*., 2017). Unlike other tyrosine kinase growth factor receptors, tumours have not been found to harbour activating mutations in IGF1R, not even as a resistance mechanism in response to IGF1R‐targeted therapies.

## Role of IGF System in Chemoresistance

IGF1R activation has been implicated in resistance to both chemical and radiation based therapies. Investigations in several different tumour types have revealed increased IGF activity in chemoresistant tumours and shown that IGF1R inhibition acts as a chemosensitizer (Dallas *et al*., 2009; Eckstein *et al*., 2009; Juan *et al*., 2011; Ireland *et al*., 2016; Cao *et al*., 2017). Downstream activation of the PI3K/AKT pathway has been shown to be instrumental to the mechanism of chemoresistance in many of these studies.

IGF1R has also been found in the nucleus. Intriguingly, nuclear IGFR1 was increased in metastatic colorectal tumours compared to matched primary tumours and correlated with poor overall survival (Codony‐Servat *et al*., 2017). Nuclear translocation of IGFR1 requires ligand‐based activation of the receptor and can be blocked by IGF1R inhibitors (Aleksic *et al*., 2010). Following entry into the nucleus, IGF1R has been shown to interact with transcriptionally active regions of DNA including the proto‐oncogene *JUN* (Aleksic *et al*., 2018). It is currently unknown whether IGF1R is found or plays a role in the nuclei of TGCT cells.

Recent studies have suggested tumour‐associated cells such as tumour‐associated macrophages (TAMs) and tumour‐associated endothelial cells (TECs) may co‐operate in IGF‐mediated chemoresistance. TAMs and myofibroblasts were found to be the main sources of IGF production in pancreatic cancer (Ireland *et al*., 2016). TECs were found to keep tumourigenesis in check by secreting IGFBP7/angiomodulin, a direct IGF1R antagonist (binding to IGF1R itself and not IGF ligands) in the presence of IGF1. However, the administration of chemotherapy appears to alter this process and IGFBP7 expression is suppressed while IGF1 expression is enhanced, allowing the TECs to be converted to promoters of tumourigenicity and consequently the emergence of chemoresistance (Cao *et al*., 2017). The induction of chemotherapy itself initiates the conversion of TECs, which perhaps perceive the chemotherapeutic agent in the same way as an injury and switch their transcriptional programme in response.

IGF1R expression is also associated with a radioresistant phenotype (Turner *et al*., 1997; Yu *et al*., 2003; Chen *et al*., 2017), suggesting that it may have a role in DNA damage response and/or repair. Several different mechanisms for the involvement of IGF1R in radioresistance have been proposed. Nuclear IGF1R is known to physically interact with and phosphorylate proliferating cell nuclear antigen (PCNA), a key mediator of the DNA damage response (Waraky *et al*., 2017). A role for IGF1R has been suggested in both of the major pathways for repairing DNA double‐strand breaks, namely homologous recombination and non‐homologous end joining (Chitnis *et al*., 2014). One of the main downstream effectors of IGF1R signalling, insulin receptor substrate 1 (IRS‐1), has been shown to interact with RAD51 which localizes to the sites of double‐strand breaks and facilitates repair by homologous recombination (Trojanek *et al*., 2003). Although a link between IGF signalling in TGCT and DNA repair has not been established in TGCT, modulation of DNA repair capacity is associated with cisplatin resistance in TGCT (Kalavska *et al*., 2018).

**Figure 2:**
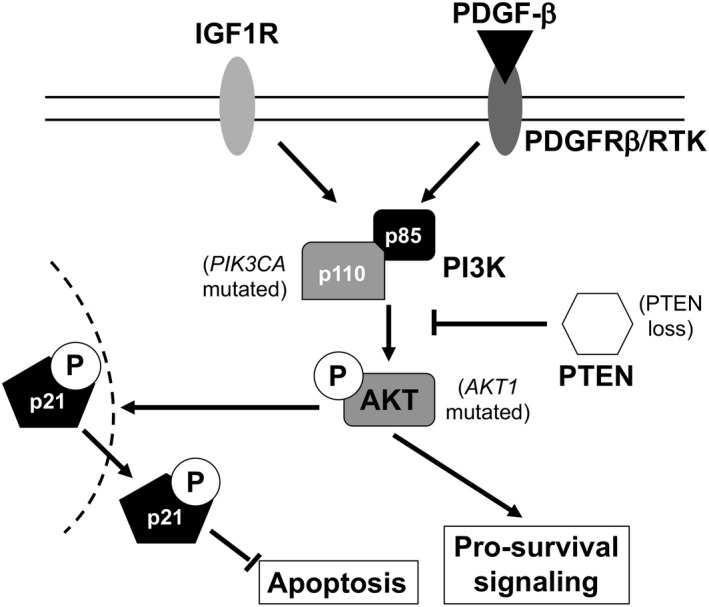
IGF signalling pathway alterations occurring in cisplatin‐resistant TGCT. Several alterations in the IGF signalling pathway have been documented in cisplatin‐resistant TGCT. *PIK3CA* and *AKT1* activating mutations have been found exclusively in resistant tumours. PTEN loss is a frequent event occurring early in TGCT evolution. Cisplatin‐resistant cells demonstrate overexpression of AKT and phospho‐AKT levels in comparison with their sensitive counterparts, which results in increased translocation of p21 to the cytoplasm where it can inhibit apoptosis. IGF1R overexpression was seen in an acquired model of resistance. Interestingly, PDGFRβ has also been shown to contribute to cisplatin resistance via PI3K/AKT signalling with overexpression of PDGF‐β.

TGCT cells are considered the paradigm of a chemosensitive tumour, readily undergoing apoptosis in response to DNA‐damaging agents such as cisplatin via a p53‐dependent pathway. The p53 response to DNA damage is intact but leads to apoptosis in preference to cell cycle arrest, in part due to very low levels of p21 in TGCT cells (Spierings *et al*., 2004). Nevertheless, although the majority of TGCT patients respond to treatment initially, a minority relapse with cisplatin refractory disease. Mutations in the *TP53* gene and amplification of its regulatory protein MDM2 are over‐represented in cisplatin‐resistant TGCT but do not explain all cases (Bagrodia *et al*., 2016). We have recently described increased IGF1R copy number, expression and activation (with increased phospho‐AKT levels) in a model of acquired cisplatin resistance (Selfe *et al*., 2018) which could subsequently be resensitized to cisplatin upon reduction of IGF1R. IGF1R hyperactivation has been specifically linked to cisplatin refractory ovarian cancer (Eckstein *et al*., 2009). There is additional evidence signifying a role for the AKT pathway in platinum‐resistant TGCT. Inhibition of AKT can restore sensitivity to cisplatin‐resistant TGCT cells by re‐localizing p21 from the cytoplasm to the nucleus (Koster *et al*., 2010), while PIK3CA and AKT1 mutations are exclusively found in cisplatin‐resistant tumours (Feldman *et al*., 2014). Phospho‐AKT levels are significantly higher in cisplatin‐resistant disease compared to sensitive or untreated tumours (Juliachs *et al*., 2014). Copy number gain and concomitant overexpression of AKT1 is a frequent event in intracranial germ cell tumours, which, although clinically and histologically similar to gonadal germ cell tumours, are more likely to be refractory to treatment (Wang *et al*., 2014). Figure 2 summarizes the key alterations in IGF signalling that have been observed in cisplatin‐resistant TGCT.

## Therapeutic Targeting of the IGF System in Cancer

IGF1R appears to represent an ideal therapeutic target in many cancers; it is expressed on the cell surface, possesses enzymatic activity and has a role in many tumourigenic processes. Two principal classes of inhibitor were initially used in a trial setting: monoclonal antibodies against IGF1R (mAbs) and small molecule tyrosine kinase inhibitors of IGF1R (TKIs). These inhibitors to IGF1R were very enthusiastically explored in many clinical trials as single agents two decades ago. Outcomes in these trials were extremely disappointing due to an overall infrequency of objective responses and a lack of any accurate predictive biomarkers of response. The most promising patient subgroup who might benefit from IGF1R‐targeted therapy are Ewing's sarcoma patients, where a minority have sustained durable responses lasting several years without major side effects (Anderson *et al*., 2016). The inability to select which patients are likely to respond has severely hampered efforts to employ IGFR1‐targeted agents in mainstream treatment.

Several explanations have been proposed to explain the lack of efficacy of IGF1R mAbs and TKIs (reviewed in Simpson *et al*., 2017). There is a large amount of crosstalk between the signalling pathways of IGF1R and other RTKs such as EGFR, ERBB2 and PDGFR (Browne *et al*., 2011; Liu *et al*., 2014). Cancer cells may therefore be able to circumvent IGF1R inhibition by upregulating another or several RTKs. As a corollary to this, it is also true that upregulation of IGF1R signalling can act as an escape mechanism in response to inhibitors of other RTKs (Ma *et al*., 2016; Almiron Bonnin *et al*., 2017; Li *et al*., 2017). The IGF1R mAbs will not prevent signalling via IGF2 binding to the INSR‐A receptor, which would be another route to evade IGF1R inhibition; indeed, increased phospho‐INSR levels have been observed in response to an EGFR inhibitor in colorectal cancer cells (Jones *et al*., 2006). IGF1R TKIs block activation of both homotetrameric and heterotetrameric INSR and IGF1R due to the similarity of the kinase regions in both proteins, and this raises the potential problem of dose limitation in order to prevent glucose metabolism being adversely affected. A newer generation of therapeutic antibodies against IGF ligands should avoid both of these pitfalls by allowing insulin to signal normally and preventing IGF2 from activating the INSR‐A receptor. Targeting the ligands instead of the receptors should also prevent resistance through downregulation of inhibitory IGF binding proteins such as IGFBP2 as seen in rhabdomyosarcoma cells (Kang *et al*., 2014).

A major hurdle in the use of anti‐IGF1R therapies in the clinic has been the lack of suitable predictive biomarkers. There is conflicting evidence as to whether expression of IGF1R itself, as opposed to activated IGF1R (phospho‐IGF1R), identifies patients that would benefit from IGF1R‐targeted agents (Cao *et al*., 2008; Kurmasheva *et al*., 2009; Zha *et al*., 2009; Cao *et al*., 2014b). Expression of IGF1R or even presence of activated IGF1R does not always signify cells that are susceptible to IGF1R inhibition. This is seen in the case of the seminoma cell line, TCAM2 (Selfe *et al*., 2018), which despite comparatively high basal levels of activated IGF1R among TGCT cell lines was among the least responsive to an IGF1R TKI. Expression of other components of the IGF axis such as IRS2 and IGFBP5 has been shown to be important for determining sensitivity to an IGF1R mAb (Pavlicek *et al*., 2013). Exclusive nuclear IGF1R correlated with a better outcome in sarcoma patients treated with an IGF1R mAb (Asmane *et al*., 2012), indicating that nuclear staining in the absence of cytoplasmic IGF1R may be a useful biomarker; however, this study had small numbers of patients.

In order to exploit the anti‐tumourigenic responses to INSR/IGF1R inhibition seen in preclinical experiments, current investigations are concentrating on combinatorial studies using IGF inhibitors. Combination with other RTK TKIs is used as a means of reducing the emergence of resistance or in addition to either standard chemotherapeutic agents or radiotherapy utilizing their properties as chemo‐ and radiosensitizers, respectively (McDermott *et al*., 2017; Schaffrath *et al*., 2017). Given the importance of the PI3K/AKT pathway in TGCT, simultaneous multiple targeting of this pathway may be effective in TGCT patients by combining IGF1R inhibition with other inhibitors of this pathway. IGF1R inhibition used in conjunction with standard chemotherapy regimens may be effective in some TGCT patients with cisplatin‐resistant disease. Initial clinical testing of this hypothesis would likely involve refractory patients for whom existing treatment options were limited or unavailable. Attempting to resensitize patients to cisplatin at an earlier stage would however be potentially more effective than in the heavily pre‐treated cases where multiple genetic events may have had time to occur and establish resistance by different mechanisms. Careful selection of cases may also be important as if *PIK3CA* or *AKT1* mutations are driving resistance, inhibition of the IGF signalling pathway should take place downstream of these. The molecular pathways that allow IGF1R inhibitors to act as chemo‐ or radiosensitizers are not yet fully understood. Identifying these mechanisms and studying their interaction with the deficiencies in DNA repair in TGCT cells will be necessary in order to exploit the full benefit of targeting the IGF axis. The mTOR inhibitor, everolimus, has shown limited efficacy in two phase‐II studies of unselected TGCT patients with refractory disease (Mego *et al*., 2016; Fenner *et al*., 2019). This may be due to the pro‐oncogenic effects of INSR/IGF1R being at least in part independent of the PI3K/AKT pathway downstream of mTOR or that IGF‐targeted therapies must be combined with DNA damaging agents to achieve clinical utility in TGCT.

## Concluding Comments

Primordial germ cells, the likely precursor of TGCT, require IGF1R signalling for correct migration to the genital ridge, and the IGF system has many roles in establishing and maintaining testicular function including steroidogenesis and maintaining pluripotency in spermatogonial stem cells. The IGF axis is dysregulated in many tumour types and can contribute to oncogenesis via multiple disparate mechanisms, making it an attractive therapeutic target. The lack of mutations found in IGF proteins in cancer may hint that INSR/IGF1R signalling is not a key driver in many tumours, and together with cross talk between pathways, this could explain the lack of efficacy seen in clinical trials using several different types of IGF1R‐targeted agent. However, there are multiple lines of evidence to suggest that cancers can use the INSR/IGF1R pathway as a resistance mechanism to other treatments and that IGF1R inhibition can augment responses to standard chemo‐ and radiotherapy. The clinical utility of blocking this pathway may therefore lie in combining newly designed IGF ligand‐targeted therapies with existing or new treatments. TGCT cells commonly exhibit aberrant IGF axis activation through elevated IGF1R activity (Selfe *et al*., 2018) and/or increased IGF2 expression through loss of imprinting (Van Gurp *et al*., 1994). We have shown that cells with high levels of IGF1R activation are vulnerable to IGF1R inhibition. Cisplatin resistance, the major cause of mortality in TGCT, may be impacted by including IGF1R inhibition.
